# A prediction model for metachronous colorectal cancer: development and validation

**DOI:** 10.1093/jnci/djaf191

**Published:** 2025-07-16

**Authors:** Ye Zhang, Amalia Karahalios, Aung Ko Win, Enes Makalic, Alex Boussioutas, Daniel D Buchanan, Stephanie L Schmit, N Jewel Samadder, Finlay A Macrae, Mark A Jenkins

**Affiliations:** Center for Epidemiology and Biostatistics, Melbourne School of Population and Global Health, University of Melbourne, Parkville, VIC, Australia; University of Melbourne Center for Cancer Research, Victorian Comprehensive Cancer Center, University of Melbourne, Parkville, VIC, Australia; Center for Epidemiology and Biostatistics, Melbourne School of Population and Global Health, University of Melbourne, Parkville, VIC, Australia; Center for Epidemiology and Biostatistics, Melbourne School of Population and Global Health, University of Melbourne, Parkville, VIC, Australia; University of Melbourne Center for Cancer Research, Victorian Comprehensive Cancer Center, University of Melbourne, Parkville, VIC, Australia; Genomic Medicine and Family Cancer Clinic, Royal Melbourne Hospital, Parkville, VIC, Australia; Center for Epidemiology and Biostatistics, Melbourne School of Population and Global Health, University of Melbourne, Parkville, VIC, Australia; Department of Data Science and AI, Faculty of Information Technology, Monash University, Clayton, VIC, Australia; Department of Medicine, Royal Melbourne Hospital, University of Melbourne, Parkville, VIC, Australia; Department of Gastroenterology, The Alfred, Monash University, Melbourne, VIC, Australia; University of Melbourne Center for Cancer Research, Victorian Comprehensive Cancer Center, University of Melbourne, Parkville, VIC, Australia; Genomic Medicine and Family Cancer Clinic, Royal Melbourne Hospital, Parkville, VIC, Australia; Colorectal Oncogenomics Group, Department of Clinical Pathology, Melbourne Medical School, The University of Melbourne, Parkville, VIC, Australia; Genomic Medicine Institute, Cleveland Clinic, Cleveland, OH, USA; Population and Cancer Prevention Program, Case Comprehensive Cancer Center, Cleveland, OH, USA; Mayo Clinic Comprehensive Cancer Center, Mayo Clinic, Phoenix, AZ, USA; Department of Medicine, Royal Melbourne Hospital, University of Melbourne, Parkville, VIC, Australia; Department of Colorectal Medicine and Genetics, University of Melbourne, Royal Melbourne Hospital, Parkville, VIC, Australia; Center for Epidemiology and Biostatistics, Melbourne School of Population and Global Health, University of Melbourne, Parkville, VIC, Australia; University of Melbourne Center for Cancer Research, Victorian Comprehensive Cancer Center, University of Melbourne, Parkville, VIC, Australia

## Abstract

**Background:**

Being able to estimate the risk of metachronous disease in a patient with colorectal cancer (CRC) could enable risk-appropriate surveillance. The aim of this study was to develop a risk-prediction model to estimate individual 10-year risk of metachronous disease following a CRC diagnosis.

**Methods:**

A population-based cohort of patients with CRC was recruited soon after diagnosis between 1997 and 2012 from the United States, Canada, and Australia. Cox regression with the least absolute shrinkage and selection operator penalization was used to identify factors that predicted the risk of a new primary CRC diagnosed at least 1 year after the initial CRC diagnosis. Potential predictors included demography, anthropometry, lifestyle factors, comorbidities, personal and family cancer history, medication use, age at diagnosis, and pathological features of the first CRC. Internal validation through bootstrapping was used to evaluate the discrimination and calibration.

**Results:**

We included 6085 CRC cases; 138 (2.3%) of these cases were diagnosed with metachronous disease over a median of 12 years (IQR = 5-17 years). Metachronous CRC risk was predicted by body mass index; smoking status; level of physical activity; family history of cancer and synchronous CRC; stage, grade, histological type, and DNA mismatch repair status; and age at diagnosis of the first CRC. The model was valid with a C statistic of 0.65 (95% CI = 0.63 to 0.68) and a calibration slope of 0.873 (SD = 0.087).

**Conclusions:**

Metachronous CRC can be predicted with reasonable accuracy using a prediction model that consists of clinical variables collected as part of routine practice.

## Introduction

Colorectal cancer (CRC) is the third-most commonly diagnosed cancer in the world.^1^ Approximately two-thirds of patients diagnosed with CRC survive to 5 years after their CRC diagnosis.^2^ This survival rate has been increasing because of improvements in treatment and early detection^1^ and has resulted in an increase in the number of patients who survive to 5 years and beyond. During this period, however, a metachronous CRC may develop.^3^ Metachronous CRC is defined as a new primary cancer of the colorectum that is not a recurrence, spread, or metastasis of the initial CRC.^4^ There are varying reports of the cumulative incidence of metachronous CRC, from 6.3% over 18 years to 30.0% over 41 years of follow-up.^5^^,^^6^

The principal method for the prevention and early detection of metachronous CRC is surveillance of the remaining colorectum by colonoscopy following diagnosis and treatment of the first CRC.^7^^,^^8^ If the individual risk of metachronous CRC could be estimated, it could have important clinical implications for surveillance, including the recommended frequency of colonoscopies of the remaining bowel. Currently, however, it is difficult to identify which CRC survivors are at increased risk of developing metachronous CRC because only a few factors have been identified as being associated with the risk of metachronous CRC. Systematic reviews^9-11^ report that the risk of metachronous CRC or advanced neoplasia is associated with patient age of diagnosis, the presence of synchronous adenomas or CRC, and the proximal location of the first CRC. There is no evidence that any lifestyle factors studied are associated with the risk of metachronous CRC or metachronous advanced neoplasia.

Several risk-prediction models have been created to identify patients at high metachronous CRC risk, but all have major limitations. They predict advanced neoplasia, the vast majority of which will be adenomas,^12-14^ or they are applicable only to patients whose first diagnosis was an adenoma rather than CRC.^15^ They are mostly based on characteristics of the adenoma and do not include other factors, such as lifestyle factors (eg, smoking status, alcohol consumption, physical activity) and comorbidities (eg, diabetes).^12-15^ Further, these models can only categorize the patients into risk category groups and do not provide absolute risks or a formula to calculate individual absolute risks of metachronous CRC.^15-17^ Our aim was to develop and validate a risk-prediction model to estimate the risk of metachronous CRC for people who survived primary CRC.

## Methods

This description of the risk-prediction model is based on the Transparent Reporting of a multivariable prediction model for Individual Prognosis Or Diagnosis guidelines.^18^ This study received ethics approval from the University of Melbourne Medicine and Dentistry Human Ethics Sub-Committee (ethics ID 13094). Informed consent was obtained from all participants.

### Data sources

All data used to develop the prediction model were obtained from the Colon Cancer Family Registry, a multicenter study that recruited a cohort of more than 8000 participants soon after their first diagnosis of CRC from population-based cancer registries, weighted toward younger-onset CRC diagnoses between 1997 and 2012 from the United States, Canada, and Australia.^19^ All participants were asked to complete the same questionnaire at baseline, which elicited detailed information about their cancer history and CRC risk factors, including demography, lifestyle, diet, screening, medication, and family history of cancer. Every 4-5 years, participants or relatives (if the participant was deceased) were asked for any updates on their cancer history. Colorectal cancer diagnoses and the age at diagnosis were verified using pathology reports, medical records, cancer registry reports, and death certificates (see Supplementary Methods for additional details).

### Participants

For this analysis, participants were restricted to individuals older than 18 years of age at recruitment who were diagnosed with their first CRC within 2 years before recruitment. Participants were excluded if they had a total resection of the colon and rectum, their first diagnosis was cancer of the appendix, data for all candidate pathological predictors were missing, or they had Lynch syndrome (a germline pathogenic variant in *MLH1, MSH2, MSH6, PMS2*, or *EPCAM*) or MUTYH attenuated polyposis (a germline pathogenic variant in *MutYH*) (see Supplementary Methods for additional details).

### Outcome definition

Metachronous CRC was defined as a new primary CRC at a different anatomical site from the first CRC that was diagnosed more than 1 year after the first CRC. Metachronous CRC was ascertained through pathology reports, medical records, cancer registry reports, and death certificates from each study site.

### Candidate predictors

Candidate predictors were obtained from the baseline questionnaire, clinical medical reports, and pathological reports of the first CRC. Potential factors for inclusion in the risk-prediction model were cases identified through previous systematic reviews^9-11^ as being associated with the risk of metachronous CRC. A complete list of candidate predictors is provided in Table 1 (see Supplementary Methods for definitions of candidate predictors).

**Table 1.** List of candidate predictors to be evaluated in the risk-prediction model.

Predictor	Variable type	Unit of measurement or category
Age at diagnosis of first CRC	Demographics factors	y
Sex	Demographics factors	Male/female
BMI	Anthropometric factors	kg/m^2^
Smoking status	Lifestyle factors	Never, former, or current smoker
Alcohol consumption	Lifestyle factors	Per 14 g/d
Physical activity	Lifestyle factors	Nonvigorous or vigorous activity
Synchronous CRC at diagnosis	Pathological factors	Yes or no
Location of initial CRC	Pathological factors	Proximal, distal, or rectum
Stage of initial CRC	Pathological factors	I, II, III, or IV
Grade of initial CRC	Pathological factors	Well/moderately or poorly differentiated
Histology type of initial CRC	Pathological factors	Adenocarcinoma or mucinous/signet ring cell carcinoma/undifferentiated
Mismatch repair status of initial CRC	Pathological factors	Mismatch repair deficient or proficient
Diabetes	Clinical comorbidities	Yes or no
Extracolonic cancer history	Clinical comorbidities	Yes or no
Aspirin intake	Medications	Yes or no
Ibuprofen intake	Medications	Yes or no
First-degree family history of CRC	Family history	Yes or no

Abbreviations: BMI = body mass index; CRC = colorectal cancer.

### Statistical analysis

#### Missing data and predictor selection

Missing data were imputed using random forests from the package *missForest* in R (R Foundation for Statistical Computing), assuming values missing at random. Using the *missForest* algorithm, 5 imputed datasets were created and averaged using Rubin rules.^20^ We fitted a Cox regression model with the Least Absolute Shrinkage and Selection Operator (LASSO) penalization,^21^ including all the candidate predictors from Table 1. The *glmnet* package in R was used to fit the LASSO-Cox regression model (see Supplementary Methods for additional details).

#### Model development

The flexible parametric survival model was fitted using the Royston-Parmar approach^22^ to estimate the coefficients of each potential predictor (ie, the strengths of the association between the predictors and metachronous CRC). For this model, the observation time metric started at the age of diagnosis of the initial CRC and ended at the age of diagnosis of metachronous CRC, last contact, or death, whichever occurred earliest (see Supplementary Methods for additional details).

#### Apparent performance and internal validation

The performance of the fitted model applied to the entire dataset was assessed using measures of discrimination and calibration. Discrimination was measured using the Harrell C statistic (95% confidence interval [CI]),^23^ which is the proportion of all patient pairs in which the predicted outcome and observed outcome are concordant.^23^ A C statistic value of 0.5 represents chance, and a value of 1 represents perfect discrimination. The calibration performance was assessed by the calibration slope. A value of 1 indicates that the predicted risk equals the observed risk across all individuals in the same development dataset. A slope less than 1 indicates that the model is overfitted. Internal validation was performed to obtain optimism-adjusted performance statistics using bootstrapping (see Supplementary Methods for additional details).

## Results

### Cohort characteristics

**Figure 1.**
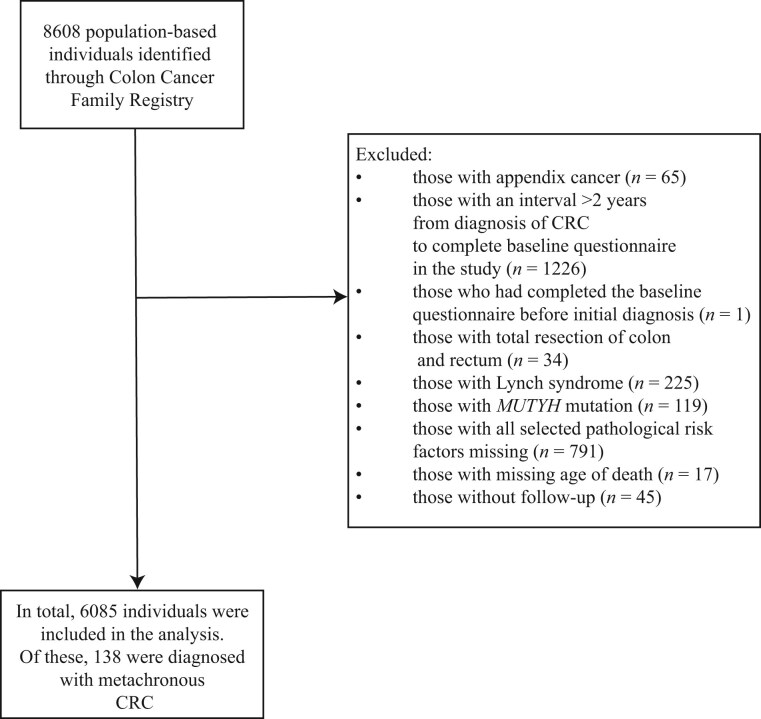
Flowchart of participants in the Colon Cancer Family Registry. CRC = colorectal cancer.

In total, 8608 CRC cases were recruited from the Colon Cancer Family Registry between 1998 and 2012. After excluding ineligible participants based on exclusion criteria, 6085 persons were included in the analysis (Figure 1). Most of the participants (*n* = 3885 [63.8%]) were recruited from the United States, and more than 75% (*n* = 4633) of individuals identified their race as White (Table S1). The summary data for each candidate predictor for the eligible participants are provided in Table 2. The proportion of missing data for each variable is shown in Table S2. Table S3 presents the distribution of baseline characteristics and candidate predictors by completeness of pathological data. The distributions were similar between the 2 groups, but missingness was associated with study site rather than with the data themselves, indicating that it was not dependent on the missing values. There was a similar proportion of male individuals (*n* = 3098 [50.9%]) and female individuals (*n* = 2987 [49.1%]). The median age of diagnosis of the first CRC for these individuals was 54 years (IQR = 46-65 years). The majority of first CRCs occurred in the distal colon (*n* = 2491 [40.9%]), followed by the proximal colon (*n* = 2114 [34.7%]) and rectum (*n* = 1480 [24.3%]).

**Table 2.** Characteristics of candidate predictors in participants.

Candidate predictors	Value
**Demographic factors**	
Age at diagnosis of initial CRC, median (IQR), y	54 (46-65)
Sex, No. (%)	
Male	3098 (50.9)
Female	2987 (49.1)
**Anthropometric factors**	
BMI, median (IQR)	26.6 (23.5-30.3)
Missing, No. (%)	96 (1.6)
**Lifestyle factors**	
Smoking status, No. (%)	
Never	2625 (43.1)
Former	2219 (36.5)
Current	1065 (17.5)
Missing	176 (2.9)
Alcohol consumption, No. (%)	
Nondrinker or <1 serving/wk	3650 (60.0)
≥1 serving/wk but ≤1 serving/d	1596 (26.2)
>1 serving/d	320 (5.3)
Missing	519 (8.5)
Physical activity, No. (%)	
Less vigorous activity	1687 (27.7)
Vigorous activity	1943 (31.9)
Missing	2455 (40.4)
**Pathological factors**	
Synchronous CRC at diagnosis, No. (%)	
No	5599 (92.0)
Yes	197 (3.2)
Missing	289 (4.8)
Location of initial CRC, No. (%)	
Distal colon	2491 (41.0)
Proximal colon	2114 (34.7)
Rectum	1480 (24.3)
TNM stage of initial CRC, No. (%)	
I	1047 (17.2)
II	1252 (20.6)
III	1434 (23.6)
IV	575 (9.4)
Missing	1777 (29.2)
Grade of initial CRC, No, (%)	
Well to moderately differentiated	4248 (69.8)
Poorly differentiated	960 (15.8)
Missing	877 (14.4)
Histologic type of initial CRC, No. (%)	
Adenocarcinoma, not otherwise specified	5159 (84.8)
Mucinous/signet ring cell carcinoma or undifferentiated	922 (15.1)
Missing	4 (0.1)
Mismatch repair status of initial CRC, No. (%)	
Proficient	4825 (79.3)
Deficient	573 (9.4)
Missing	687 (11.3)
**Clinical comorbidities**	
Diabetes, No. (%)	
No	5350 (87.9)
Yes	715 (11.8)
Missing	20 (0.3)
**Medications**	
Aspirin intake, No. (%)	
No	4358 (71.6)
Yes	1683 (27.7)
Missing	44 (0.7)
Ibuprofen intake, No. (%)	
No	5003 (82.2)
Yes	1000 (16.4)
Missing	82 (1.4)
**Others**	
First-degree family history of CRC, No. (%)	
No	4854 (79.8)
Yes	1231 (20.2)
Extracolonic cancer history, No. (%)	
No	5733 (94.2)
Yes	352 (5.8)

Abbreviations: BMI = body mass index; CRC = colorectal cancer.

### Risk of metachronous CRC

Of 6085 patients with CRC, 138 (2.3%) developed metachronous CRC, with an incidence rate of was 2.0 (95% CI = 1.7 to 2.4) per 1000 person-years. The median age at diagnosis of metachronous CRC was 69 years (IQR = 56-77 years). The median time to develop metachronous CRC was 8 years (IQR = 4-13 years).

### Predictor selection

The Cox regression model with LASSO penalization selected 11 variables for the final model: age at first CRC diagnosis, body mass index (BMI; log-transformed), smoking status, level of physical activity within 2 years of first CRC diagnosis, first-degree family history of CRC, extracolonic cancer history, and pathology features of the first CRC (ie, synchronous CRC, stage, grade, histological type, and mismatch repair status). The cross-validation results of LASSO for the final model are shown in Figure S1. The LASSO coefficients of the selected predictors are shown in Table S4.

### Model development

The flexible parametric model with 3 knots was fitted with the predictors selected using Cox regression with the LASSO penalization. Based on the patients’ characteristics, the survival probability (ie, the probability of not developing metachronous CRC) at time point *t* is


$$S_{\left(t\right)}=S_{0}{\left(t\right)}^{\;\text{exp}\;}[(\beta_{1}´X_{1}+\cdots \ldots +\beta_{n}´X_{n})]$$

where β_1_ – β_n_ are the coefficients for predictors shown in Table 3, *X1 – X_n_* are the predictor values for the participants, and *S_0_* (*t*) is the value of the baseline survival function at time *t* shown in Table S5. Written formally, the equation used to derive individual risk of metachronous CRC over time is shown in Box S1.

**Table 3.** Coefficients of predictors for metachronous CRC in the flexible parametric model using a dataset with missing values imputed.

Selected predictor	Flexible parametric model coefficient (95% CI)
Age at diagnosis of initial CRC, centered at a mean of 55 y, 7	0.014 (‒0.002 to 0.030)
Recent BMI (log-transformed) centered at a mean of 3.3	0.800 (‒0.053 to 1.653)
Former smoker (vs nonsmoker)	0.187 (‒0.195 to 0.568)
Current smoker (vs nonsmoker)	0.557 (0.095 to 1.018)
Synchronous CRC at diagnosis (vs no)	1.268 (0.689 to 1.848)
Stage II (vs stage I) disease	‒0.331 (‒0.774 to 0.113)
Stage III (vs stage I) disease	‒0.255 (‒0.717 to 0.267)
Stage IV (vs stage I) disease	1.122 (0.581 to 1.661)
Poorly or undifferentiated grade of initial CRC (vs well to moderate differentiated grade)	‒0.821 (‒1.443 to ‒0.200)
Mucinous/signet ring cell carcinoma or undifferentiated (vs adenocarcinoma)	‒0.260 (‒0.768 to 0.245)
Mismatch repair deficient (vs mismatch repair proficient)	0.919 (0.469 to 1.369)
First-degree family history of CRC (vs no)	0.438 (0.072 to 0.803)
Vigorous physical activity (vs less vigorous activity)	‒0.160 (‒0.512 to 0.193)
Extracolonic cancer history (vs no)	0.358 (‒0.292 to 1.009)

Abbreviations: BMI = body mass index; CI = confidence interval; CRC = colorectal cancer.

### Performance of the prediction model

**Figure 2.**
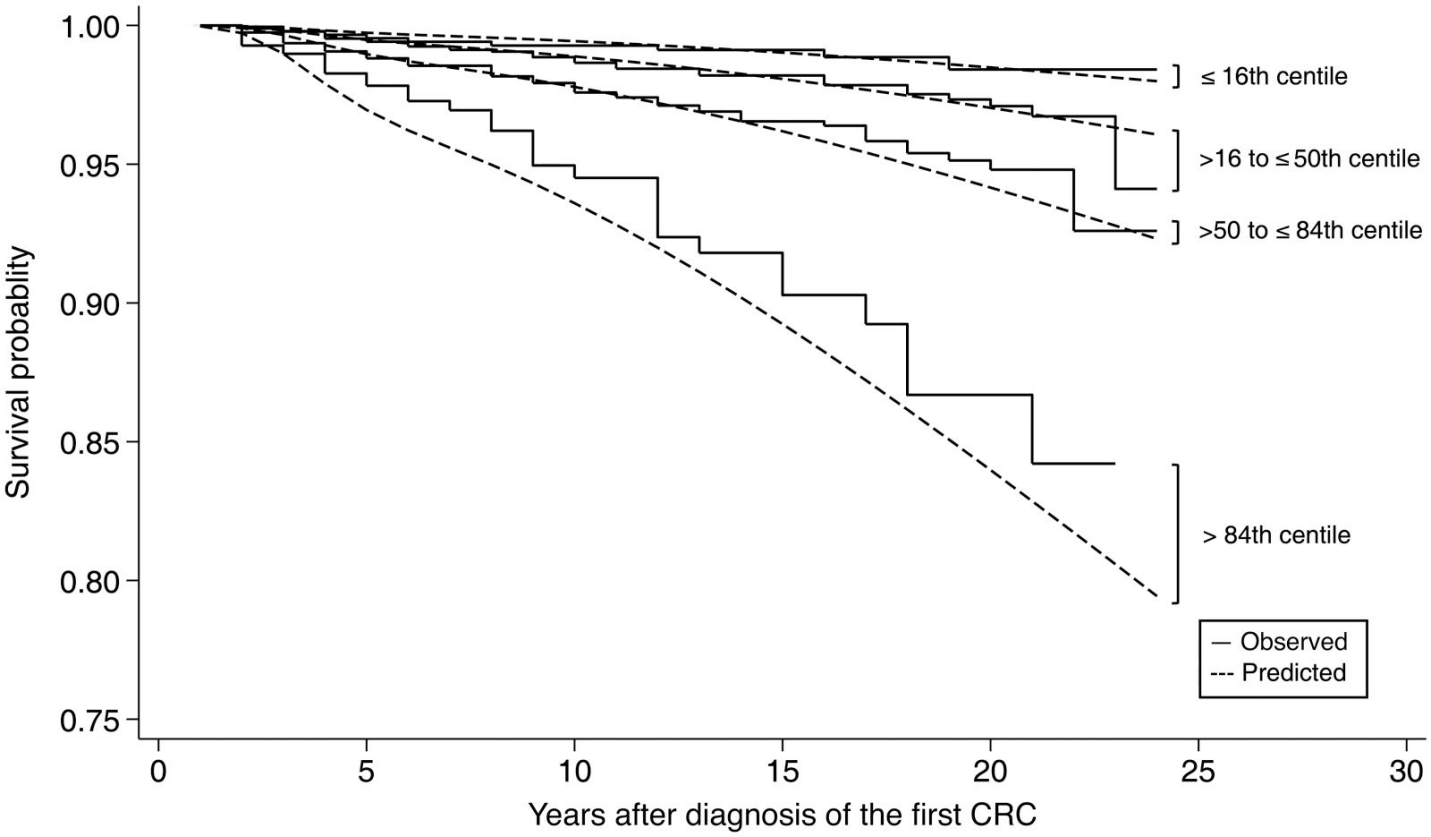
Mean survival curves for groups of the prognostic index compared with their observed Kaplan-Meier survival curves. CRC = colorectal cancer.

The C statistic of the final model was 0.68 (95% CI = 0.64 to 0.73). The predicted risk of metachronous CRC was divided into 4 groups defined by the 16th, 50th, and 84th centiles based on the percentage distribution of observations in the optimal grouping for 4 to retain as much information about linear predictors as possible.^24^ The agreement assessed by comparing the observed and predicted number of metachronous CRCs was high for people after a first diagnosis of CRC across these percentiles of estimated risk (Table 4). The comparison of predicted mean survival probability (ie, the probability of not developing metachronous CRC) curves for 4 risk groups and their observed Kaplan-Meier curves is shown in Figure 2. Overall, our model showed an excellent ability to distinguish between patients with low-risk and high-risk CRC. The agreement was generally excellent, except for the highest-risk group, where the observed absolute risk of metachronous CRC was lower than predicted from the model.

**Table 4.** Number of metachronous CRC cases within groups of predicted risk.

Centile of predicted risk^a^	Patients with CRC, No.	Patients with metachronous CRC, No. (%)
≤16th	973	9 (0.9)
>16th to ≤50th	2069	38 (1.8)
>50th to ≤84th	2070	57 (2.8)
>84th	973	34 (3.5)

Abbreviation: CRC = colorectal cancer.

a Defined by the 16th, 50th, and 84th centiles of the predicted risk of metachronous CRC based on the percentage distribution of observations in the optimal grouping for 4.

### Internal validation

The risk-prediction model had an optimism of 0.030 (SD = 0.023) in the C statistics and 0.127 (SD = 0.087) in the calibration slope. This minimal optimism adjustment to the C statistic indicates good internal performance in terms of discrimination. The optimism-adjusted Harrell C statistic of the model was 0.65 (95% CI = 0.63 to 0.68). The optimism calibration was reduced to 0.873, however, which indicates that the spread of predicted risks is extreme (ie, too high for individuals at high risk), as shown in Figure 2. The calibration plot is shown in Figure S2.

### Application of the developed model

To demonstrate how each risk factor affected the predicted risk of metachronous CRC, we provided 2 examples of the predicted risks 10 years after the first CRC for individuals who differ by these risk factors. The risk factors and predicted values of these 2 example individuals are provided in Table S6. The risk calculation can be readily modified to account for different times because the first CRC by replacing the baseline survival value with the corresponding value from the desired time point, as provided in Table S5.

## Discussion

We have developed and validated a prediction model for metachronous CRC that could feasibly help health professionals tailor surveillance strategies. This model includes demography, lifestyle, features of first CRC, and cancer history factors. It has modest discrimination and reasonable calibration and can estimate individual risk of metachronous CRC up to 20 years after the diagnosis of a first CRC.

### Strengths and limitations

We used the world’s largest prospective cohort of 6085 patients with CRC recruited from population-based registries for a median of 12 years. A wide range of candidate predictors were assessed during model development. All cases of CRC had undergone a standardized pathology review to ensure consistency of classification across recruiting sites. We used a conservative definition of metachronous CRC as diagnosed at least 1 year after the first CRC diagnosis to reduce any misclassification of synchronous tumors as metachronous. This study also has limitations. No information was available on the quality of surveillance colonoscopies after the first CRC; therefore, it was not possible to assess the likelihood that some metachronous CRCs were missed or the degree to which metachronous CRCs were prevented by polyp removal. The extent of resection and treatment for the first CRC was not included as a predictor due to limited availability and completeness of data. In addition, because we did not have information regarding potential differences in colonoscopy surveillance by country, we could not assess compliance or adenoma detection rates. Although there was a large number of missing values for some of the predictors, we were able to provide their values using imputation, resulting in reasonable predictive performance.^25^ As participants with inherited cancer syndromes were excluded, the findings are unlikely to be generalizable to patients with CRC and Lynch syndrome.

### Comparison with existing evidence

Our finding that age at diagnosis of initial CRC, BMI, smoking status, presence of synchronous CRC, mismatch repair–deficient tumor status of first CRC, and first-degree family history of CRC are predictors for metachronous CRC is consistent with previous studies.^13^^,^^16^^,^^17^ We were unable, however, to replicate the finding that location of the first CRC (proximal vs distal) and diabetes are also predictors of metachronous CRC.^16^^,^^26^^,^^27^ Our findings that physical activity level within 2 years before the diagnosis of the first CRC, TNM stage of the first CRC, and extracolonic cancer history contribute to the prediction of metachronous CRC are novel.

We used LASSO regression to develop our prediction model because we believe that it has advantages over the commonly used stepwise selection of risk-prediction studies. This method has been criticized for creating overly optimistic regression estimates^28^ (ie, it overfits the data). In contrast, LASSO penalization does not take the *P* value into account during the model selection step of the process. Instead, it uses a tuning parameter *λ*, determined by cross-validation, to penalize the number of predictors in the model,^21^^,^^29^ which minimizes the mean SE of the prediction. This penalization helps increase model interpretability by eliminating irrelevant variables that are not associated with the outcome, reducing the likelihood of overfitting. In addition, LASSO penalization is an appealing method when there is a small number of outcomes per variable tested.^30^ It has been suggested that when using stepwise selection, 10 or more events (ie, metachronous cancers) are needed per variable in the prediction model to avoid overfitting.^31^ In our model of 17 tested parameters, we would require at least 170 metachronous CRC cases to justify a stepwise regression, which is more than the 138 metachronous CRCs in our data.

To improve the feasibility of our prediction model for application in clinical practice, we used variables that should be available in the medical records near the time of diagnosis of the first CRC. Accordingly, we used data collected from patients recruited soon after their CRC diagnosis. Finally, in contrast to previously published models that relied on Cox proportional hazards regression,^16^^,^^17^ our analysis used the flexible parametric survival model, which enabled us to predict the absolute risk of metachronous CRC for individuals by time since the first CRC.

### Implications for clinical practice

Our prediction model provides for individualized risks of metachronous CRC after diagnosis of the first CRC. The US Multi-Society Task Force on CRC recommends that all patients who had curative resection of CRC undergo surveillance colonoscopy at 1, 6, and 11 years after the resection of the first CRC.^8^ In Australian clinical practice, guidelines for surveillance colonoscopy recommend an initial postoperative colonoscopy at 1 year, followed by subsequent colonoscopies after 3 years, then 5 years, depending on an individualized estimate of risk.^32^ Once our model is developed into a risk tool, clinicians could estimate this personalized estimate of metachronous CRC risk and use these predictions to help guide their clinical decisions on surveillance because it can estimate personalized risk of metachronous CRC at 1, 3, 5, and 10 years after diagnosis of the first CRC. The example of risk calculation shown in Table S6 is for a patient with CRC diagnosed with stage II CRC at 64 years of age with a BMI of 3.3 who had a first-degree family history of CRC and no other risk factors. That patient’s 10-year risk of metachronous CRC is 2.5%. If this low risk is correlated with low velocity of growth, then a recommendation for a longer surveillance interval could be appropriate. In contrast, a patient with CRC diagnosed with stage III CRC at 70 years of age with a BMI of 3.6 and who is a former smoker has a predicted 10-year risk of 8.1%, which is 3 times higher than the first patient and 4 times higher than the average risk of developing metachronous CRC. The current guidelines may be too conservative for this second patient, who could benefit from more extensive colonoscopy surveillance than the first patient. Once our model has been validated in an external cohort and converted into a risk tool, it will provide clinicians with the opportunity to incorporate patient-specific, objective estimates alongside established guidelines and their clinical judgment to inform appropriate surveillance recommendations for their patients. In future research, genetic factors should be incorporated into the model to improve the effectiveness of this risk-prediction model. Risk-prediction models that incorporate genetic variables are better at discriminating between individuals at higher and lower risk of CRC than models that use age, family history, or lifestyle/environmental risk factors alone.^33^ In addition, this model should be externally validated; however, we could not identify a cohort with a sufficiently large number of patients with CRC followed prospectively over a median of 12 years.

In conclusion, using the largest CRC family cohort in the world, we developed a prediction model to estimate the personalized risk of metachronous CRC at various time points (in years) for people who survived primary CRC. Our prediction model has the potential to help clinicians make decisions about surveillance recommendations.

## Supplementary Material

djaf191_Supplementary_Data

## Data Availability

The deidentified data used in this study are available following an application to the Colon Cancer Family Registry (https://www.coloncfr.org/collaboration). Other data that support the findings of this study are available from the corresponding author upon request.
