# Supplementary material for: A prediction model for metachronous colorectal cancer: development and validation
Source: J Natl Cancer Inst. 2025 Jul 16;117(10):2082–8. doi: 10.1093/jnci/djaf191 (PMC12505132; doi:10.1093/jnci/djaf191)
Supplement: djaf191_Supplementary_Data [file djaf191_supplementary_data.zip › Clean_Supplementary Materials.pdf]

Supplementary Materials

Supplementary Methods

Data source

The Colon Cancer Family Registry is the largest family cohort study for the investigation of colorectal cancer. Participants were recruited from the Ontario Cancer Registry, the Fred Hutchinson Cancer Research Centre, Mayo Clinic, SEER Registry for State of Hawaii, University of Southern California Consortium and Victorian Cancer Registry. After completing the baseline questionnaire, attempts were made to follow-up cases every 4-5 years by telephone interview or mailed a questionnaire for updated information on their cancer history. Tumor location and histology were classified using the International Classification of Diseases for Oncology, third edition (ICO-O-3).[1] Pathology reports for the first CRC for each CRC case were reviewed by pathologists from each study center of the Colon CFR to confirm the diagnosis of CRC and to extract pathology features.[2]

Participants

The Colon Cancer Family Registry Cohort attempted to recruit participants within 2 years following their diagnosis. We restricted our inclusion to those participants as our aim was to develop prediction models for those recently diagnosed with colorectal cancer in an attempt to reduce recall error for exposures before their colorectal cancer diagnosis. To reduce the selection bias, we compared the baseline characteristics and predictors of the included participants who were missing any pathological features to those who had complete data.

Candidate predictors

Smoking status was defined as ever smoking one cigarette per day for 3 months or longer. Current smoking was indicated when persons reported smoking in the two years prior to

enrolment; former smoking was indicated when persons stopped smoking before the two years prior to enrolment. A serving was defined as a 12-ounce can/bottle of beer/cider, a 4-ounce glass of wine / 1-ounce glass of fortified wine, or a 1-ounce shot of hard liquor. Vigorous activity was defined as people taking activities (>= 6 METs) more than 1 hour per week. Synchronous CRC was defined as a CRC occurring within one year after the diagnosis of the first CRC. Categorized according to the International Classification of Diseases for Oncology, Third Edition anatomical site codes: C180, C182, C183, C184 (proximal colon); C185, C186, C187, C199, C209 (distal colon/rectum). TNM staging was classified according to the American Joint Committee on Cancer staging manual. Well and moderately differentiated were defined as tumors exhibiting at least 50% gland formation, whereas poorly differentiated were defined as tumors with <50% gland formation. Mucinous carcinoma and signet-ring carcinoma were defined as at least 50% of the tumor demonstrating a mucinous or signet-ring cell differentiation, respectively. Tumors were defined as MMR-deficient if they were microsatellite instability (MSI)-high (instability for at least 30% of the DNA markers) and/or showed loss of expression of one or more of the MMR proteins by immunohistochemistry (IHC). Tumors were defined as MMR-proficient if they were microsatellite stable (instability for 0% of the DNA markers) or MSI-low (instability for less than 30% the DNA markers)), and normal expression of all four MMR proteins by IHC.

Statistical analysis

Missing data and predictor selection

To investigate the missing mechanism, we compared the characteristics of the participants who were missing any pathological features to those who had complete data. The missing at random method assumes that the missing values in the data are related to the other (observed) variables in the data; i.e., missingness may depend on the other observed variables but not on any

unobserved variables. There is no way for us to determine whether this assumption is true. We used 10-fold cross-validation to select the optimal penalty tuning parameter λ in the LASSO penalization. During the predictor selection, age and BMI were modeled as continuous variables rather than categorical to preserve the full range of information for prediction. Additionally, BMI was log-transformed to achieve a linear relationship between BMI and the risk of metachronous CRC.

Model development

The flexible parametric survival model models the log cumulative baseline hazard using restricted cubic splines (implemented as the stpm2 package in Stata18).[3] Participants were regarded as being at risk from the time of diagnosis of the first CRC. The failure event was metachronous CRC, i.e., a new CRC occurring at least one year after the first CRC diagnosis. We chose a flexible parametric model rather than the Cox regression model because the flexible parametric model allows for the incorporation of non-linear functions of baseline hazard rate via restricted cubic splines, which are relatively easy to apply. We used the Akaike Information Criterion/Bayesian Information Criterion (AIC/BIC) to select how many knots were used to estimate the baseline hazard function. The location of the knots was set to the centiles of the distribution of uncensored log-transformed event times, which is the default location specified by the stpm2 command.

Apparent performance and internal validation

Discrimination refers to the model's ability to distinguish between patients who develop metachronous CRC during the study and those who do not.[4] Calibration describes the ability of the model to correctly estimate the absolute risk.[4] Internal validation was performed to get using bootstrapping according to the following steps. First, individuals were randomly selected

with replacements from the imputed dataset, a process that continued until a new dataset of the same size was created. This procedure was repeated 500 times to generate 500 bootstrap samples. For each of the 500 bootstrap samples, the predictor selection and modeling process was performed. Next, the measures of performance of the model (i.e., *c*-statistic and calibration slope) were calculated for each bootstrap sample (i.e., the apparent performance), and then compared with the measure of performance in the original sample (i.e., test performance) to obtain the difference between the apparent and test performance. Finally, the differences between the apparent and test performance measures from the 500 bootstrap samples were averaged to obtain a single estimate of optimism for the *c*-statistic and the calibration slope. This optimism was subsequently deducted from the original apparent performance statistics to yield optimism-adjusted performance statistics.[5]

References

1. World Health Organization. International classification of diseases for oncology (ICD-O)–3rd edition, 1st revision. 2013;

2. Jenkins MA, Hayashi S, O'Shea AM, et al. Pathology features in Bethesda guidelines predict colorectal cancer microsatellite instability: a population-based study. *Gastroenterology*. Jul 2007;133(1):48-56. doi:10.1053/j.gastro.2007.04.044

3. Royston P. Flexible parametric alternatives to the Cox model, and more. *The Stata Journal*. 2001;1(1):1-28.

4. D'Agostino RB, Nam B-H. Evaluation of the performance of survival analysis models: discrimination and calibration measures. *Handbook of statistics*. 2003;23:1-25.

5. Collins GS, Dhiman P, Ma J, et al. Evaluation of clinical prediction models (part 1): from development to external validation. *BMJ*. Jan 8 2024;384:e074819. doi:10.1136/bmj-2023-074819

Table S1 Baseline characteristics for the 6085 colorectal cancer (CRC) cases recruited from the Colon Cancer Family Registry between 1997 and 2012.

Baseline characteristics	N (%)[a]
**Colon Cancer Family Registry site**	
Sinai Health System (formerly Cancer Care Ontario)	1529 (25.1)
University of Southern California Consortium	925 (15.2)
University of Melbourne	671 (11.0)
University of Hawaii	384 (6.3)
Mayo Clinic	543 (8.9)
Fred Hutchinson Cancer Center	1882 (31.0)
University of California, San Francisco (formerly Cancer Prevention Institute of California)	151 (2.5)
**Country**	
Canada	1529 (25.1)
USA	3885 (63.9)
Australia	671 (11.0)
**Race**	
American Indian	99 (1.6)
Asian	617 (10.1)
Black	642 (10.6)
Pacific islander	15 (0.3)
White	4633 (76.1)
More than one race	66 (1.1)
Unknown	13 (0.2)

[a] n (%) presented unless otherwise stated.

Table S2 Missing proportion for each candidate predictor.

**Candidate predictors**	**Missing data (%)**[a]
Demographic factors	
**Age at diagnosis of initial CRC (years)**	
Missing	0 (0)
**Gender**	
Missing	0 (0)
Anthropometric factors	
**Body mass index (kg/m^2)**	
Missing	96 (1.6)
Lifestyle factors	
**Smoking status**	
Missing	176 (2.9)
**Alcohol consumption**	
Missing	519 (8.5)
**Physical activity**	
Missing	2455 (40.4)
Pathological factors	
**Synchronous CRC at diagnosis**	
Missing	289 (4.8)
**Location of initial CRC**	
Missing	0 (0)
**TNM stage of initial CRC**	
Missing	1777 (29.2)
**Grade of initial CRC**	
Missing	877 (14.4)
**Histologic type of initial CRC**	
Missing	4 (0.1)
**Mismatch repair status of initial CRC**	
Missing	687 (11.3)
Clinical comorbidities	
**Diabetes**	
Missing	20 (0.3)
Medications	
**Aspirin intake**	
Missing	44 (0.7)
**Ibuprofen intake**	
Missing	82 (1.4)
Others	
**First degree family history of CRC**	
Missing	0 (0)
**Extracolonic cancer history**	
Missing	0 (0)

[a] n (%) presented unless otherwise stated.

Table S3 Distribution of characteristics and predictors between individuals with and without complete pathological factors.

	Complete pathological features N=3711(%)[a]	Missing any pathological feature N=2374 (%)[a]
**Sex**		
Male	1884 (50.8)	1214 (51.1)
Female	1827 (49.2)	1160 (48.9)
**Age at diagnosis of initial CRC (years)**		
Median [interquartile range]	54 [46-65]	53 [45-65]
**Body Mass Index, kg/m^2**		
Median	26.6 [23.5-30.3]	27.0 [24.6-30.1]
Missing	37 (1.0)	59 (2.5)
**Smoking status**		
Never	1555 (41.9)	1070 (45.1)
Former smoker	1482 (39.9)	837 (35.3)
Current smoker	647 (17.4)	418 (17.6)
Missing	27 (0.8)	49 (2.1)
**Alcohol consumption, per 14g/day**		
Median [interquartile range]	1.4 [0-11.9]	1.7 [0-16.7]
Missing	319 (8.6)	200 (8.4)
**Physical activity**		
Less-vigorous activity	1084 (29.2)	603 (25.4)
Vigorous activity	1166 (31.4)	777 (32.7)
Missing	1462 (39.4)	994 (41.9)
**Diabetes**		
No	3320 (89.5)	691 (85.5)
Yes	376 (10.1)	98 (14.2)
Missing	15 (0.4)	5 (0.2)
**Aspirin intake**		
No	2664 (71.8)	1694 (71.4)
Yes	1013 (27.3)	670 (28.2)
Missing	34 (0.9)	10 (0.4)
**Ibuprofen intake**		
No	3027 (81.6)	1976 (83.2)
Yes	627 (16.9)	373 (15.7)
Missing	57 (1.5)	25 (1.1)
**First degree family history of CRC**		
No	2929 (78.9)	1925 (81.1)
Yes	782 (21.1)	449 (18.9)
**Extracolonic cancer history**		
No	3493 (94.1)	2240 (94.4)
Yes	218 (5.9)	134 (5.6)
**Colon Cancer Family Registry site**		
Sinai Health System (formerly Cancer Care Ontario)	456 (12.3)	1,069 (45.4)
University of Southern California Consortium	910 (24.5)	9 (0.5)
University of Melbourne	525 (14.1)	142 (6.1)
University of Hawaii	305 (8.2)	75 (3.3)
Mayo Clinic	515 (13.9)	24 (1.1)
Fred Hutchinson Cancer Center	862 (23.2)	1,016 (43.1)

University of California, San Francisco (formerly Cancer Prevention Institute of California)	138 (3.8)	9 (0.5)
**Race**		
American Indian	56 (1.6)	43 (1.7)
Asian	432 (11.7)	185 (7.7)
Black	586 (15.8)	56 (2.3)
Pacific islander	7 (0.2)	8 (0.2)
White	2574 (69.2)	2059 (87.2)
More than one race	50 (1.4)	16 (0.6)
Missing	6 (0.1)	7 (0.3)
**Education**		
Primary school	60 (1.7)	48 (1.9)
High school	1,308 (35.2)	856 (36.2)
Vocational training	439 (11.9)	195 (8.1)
Some college and university	820 (22.1)	584 (24.6)
Bachelor and above	1,033 (27.8)	684 (28.9)
Missing	51 (1.4)	7 (0.3)
**Annual household Income 2 years ago**		
Less than 15K	290 (7.9)	134 (5.5)
15K-30K	519 (14.0)	328 (13.8)
30K-45K	607 (16.4)	449 (18.9)
45K-70K	683 (18.4)	482 (20.3)
Over 70K	804 (21.6)	473 (20.0)
Missing	808 (21.7)	508 (21.5)

[a] n (%) presented unless otherwise stated.

Table S4 Coefficients of selected predictors by Least Absolute Shrinkage and Selection Operator (LASSO) regression using a dataset with missing values imputed.

**Selected Predictors**	**LASSO-Cox coefficient**
Age at diagnosis of initial CRC centered at mean 55 *(years)*	0.011
Recent BMI (log-transformed) centered at mean 3.3 *(kg/m^2)*	0.489
Former Smoker *(vs Non-Smoker)*	0.016
Current Smoker *(vs Non-Smoker)*	0.296
Synchronous CRC at diagnosis *(vs No)*	1.105
Stage II *(vs Stage I)*	-0.153
Stage III *(vs Stage I)*	-0.102
Stage IV *(vs Stage I)*	0.870
Poorly or undifferentiated grade of initial CRC *(vs Well to moderate differentiated grade)*	-0.593
Mucinous/signet ring cell-carcinoma or undifferentiated *(vs Adenocarcinoma)*	-0.112
MMR deficient *(vs MMR proficient)*	0.718
First degree family history of CRC *(vs No)*	0.357
Vigorous physical activity *(vs Less-vigorous activity)*	-0.107
Extracolonic cancer history *(vs No)*	0.142

LASSO=Least Absolute Shrinkage and Selection operator

Table S5 Baseline survival at various time points for CRC patients.

Time point (years)	Baseline survival, $S_0(t)$
1	0.99994
2	0.99934
3	0.99753
4	0.99487
5	0.99251
6	0.99066
7	0.98907
8	0.98750
9	0.98579
10	0.98391
11	0.98187
12	0.97969
13	0.97735
14	0.97486
15	0.97224
16	0.96948
17	0.96660
18	0.96361
19	0.96050
20	0.95729
21	0.95398
22	0.95059
23	0.94712
24	0.94357

Table S6 Calculation of the risk of metachronous colorectal cancer by 10 years using the developed model.

Predictor	Example 1			Example 2		
	Predictor values	Calculation		Predictor values	Calculation	
**Time point**	**10 years**			**10 years**		
Baseline survival		$=0.98391^{exp}$ (…			$=0.98391^{exp}$ (…	
Age at diagnosis of initial CRC-55	64	0.014 × 9	+0.126	70	0.014 × 15	+0.21
Recent BMI (log-transformed)-3.29	3.310	+0.800 × 0.010	+0.008	3.595	+0.800 × 0.295	+0.236
Former Smoker	1	+0.187	+0.187	1	+0.187	+0.187
Current Smoker	0	+0	+0	0	+0	+0
Synchronous CRC at diagnosis	0	+0	+0	1	+1.268	+1.268
Stage II	1	-0.331	-0.331	0	+0	+0
Stage III	0	+0	+0	1	-0.255	-0.255
Stage IV	0	+0	+0	0	+0	+0
Poorly or undifferentiated grade of initial CRC	0	+0	+0	0	+0	+0
Mucinous/signet ring cell-carcinoma or undifferentiated type of initial CRC	0	+0	+0	0	+0	+0
MMR deficient	0	+0	+0	0	+0	+0
First degree family history of CRC	1	+0.438	+0.438	0	+0	+0
Vigorous activity	0	+0	+0	0	+0	+0
Extracolonic cancer history	0	+0	+0	0	+0	+0
Score			=0.428			=1.646
Predicted survival by 10 years			=0.97542			=0.91931
Predicted risk of metachronous CRC by 10 years			2.5%			8.1%

Figure S1 Selection of optimal tunning parameters (lambda) from the LASSO model using 10-fold cross-validation and minimum criteria.

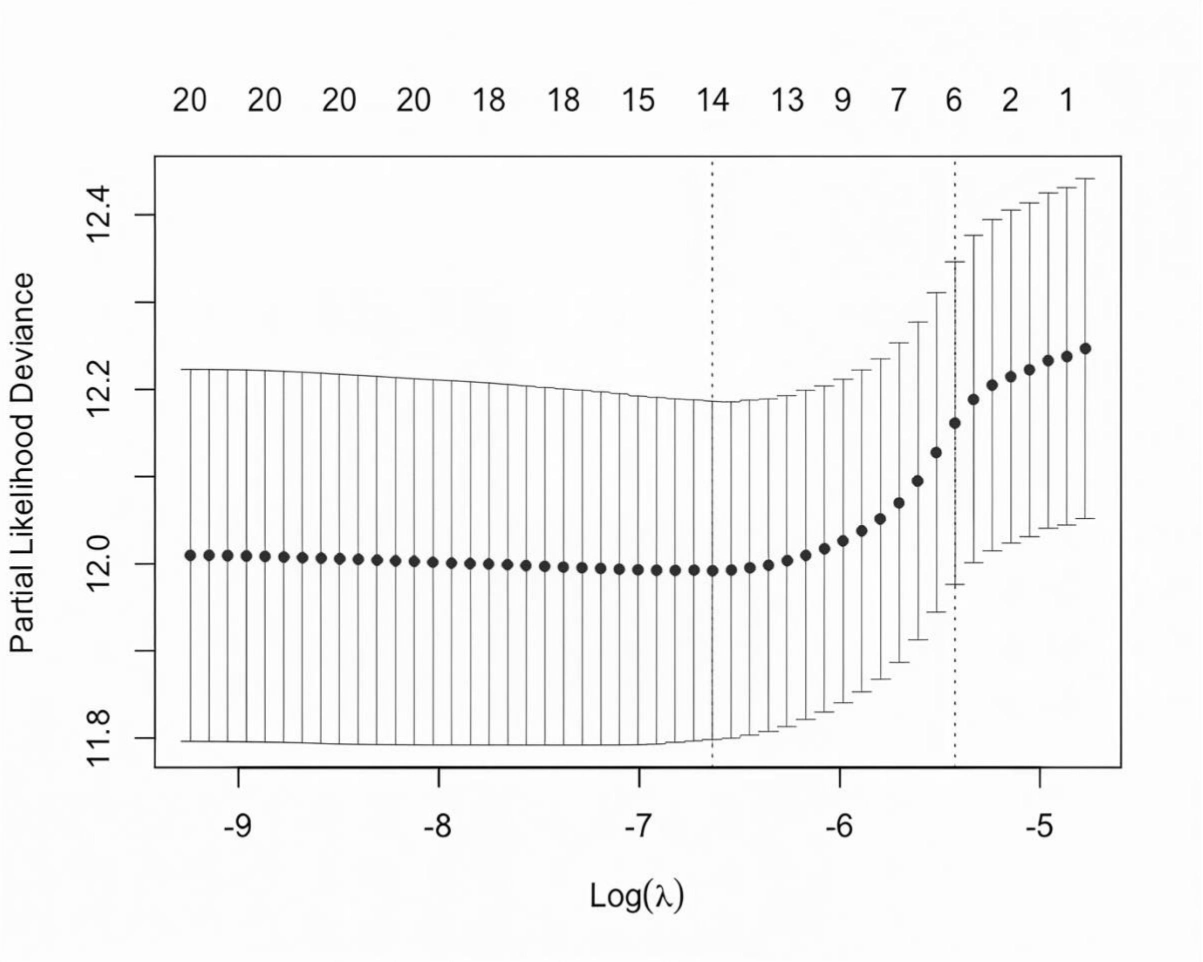

The partial likelihood deviance curve is plotted against the log(lambda). Dotted vertical lines are drawn at the optimal values using the minimum criteria and the 1-standard error of the minimum criteria (1-SE criteria). The number across the top of the figure shows the number of selected predictors.

Figure S2 The calibration plot curve for the risk of metachronous colorectal cancer 10 years after the diagnosis of the first colorectal cancer.

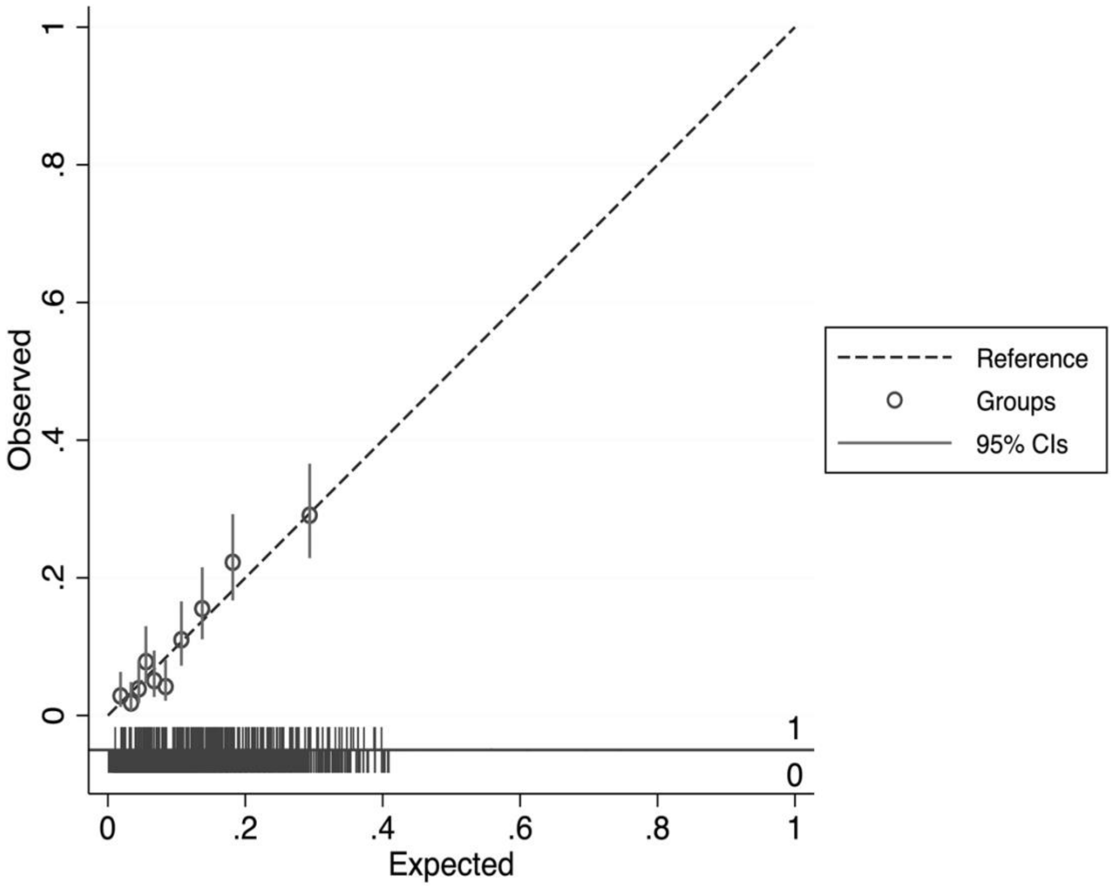

Box S1 The equation to predict individual risk of metachronous CRC over time.

P (probability of developing metachronous CRC)

$=1-S(t)$

$=1-S_0(t)^{exp}$ [(0.014× (Age at diagnosis of initial CRC - 55)

+0.800× (log-transformed recent BMI - 3.3)

+0.187 if former smoker

+0.557 if current smoker

+1.268 if the first CRC was synchronous

-0.331 if the first CRC was Stage II

-0.255 if the first CRC was Stage III

+1.122 if the first CRC was Stage IV

-0.821 if the first CRC was poorly or undifferentiated grade

-0.260 if the first CRC was Mucinous/signet ring cell-carcinoma or undifferentiated type

+0.919 if the first CRC was mismatch repair deficient

+0.438× with a First-degree family history of CRC

-0.160× if vigorous physical activity

+0.358× with extracolonic cancer history]

Where $S_0(t)$ is the value of the baseline survival function at time *t*.
